# Improvement of Contact Tracing with Citizen’s Distributed Risk Maps

**DOI:** 10.3390/e23050638

**Published:** 2021-05-20

**Authors:** Miguel Rebollo, Rosa María Benito, Juan Carlos Losada, Javier Galeano

**Affiliations:** 1 VRAIn-Valencian Research Institute for Artificial Intelligence, Universitat Politècnica de València, 46022 Valencia, Spain; 2 Complex Systems Group, Universidad Politécnica de Madrid, 28040 Madrid, Spain; rosa.benito@upm.es (R.M.B.); juancarlos.losada@upm.es (J.C.L.); javier.galeano@upm.es (J.G.)

**Keywords:** consensus, complex network, COVID, risk map, collaboration, contact tracing

## Abstract

The rapid spread of COVID-19 has demonstrated the need for accurate information to contain its diffusion. Technological solutions are a complement that can help citizens to be informed about the risk in their environment. Although measures such as contact traceability have been successful in some countries, their use raises society’s resistance. This paper proposes a variation of the consensus processes in directed networks to create a risk map of a determined area. The process shares information with trusted contacts: people we would notify in the case of being infected. When the process converges, each participant would have obtained the risk map for the selected zone. The results are compared with the pilot project’s impact testing of the Spanish contact tracing app (RadarCOVID). The paper also depicts the results combining both strategies: contact tracing to detect potential infections and risk maps to avoid movements into conflictive areas. Although some works affirm that contact tracing apps need 60% of users to control the propagation, our results indicate that a 40% could be enough. On the other hand, the elaboration of risk maps could work with only 20% of active installations, but the effect is to delay the propagation instead of reducing the contagion. With both active strategies, this methodology is able to significantly reduce infected people with fewer participants.

## 1. Introduction

The current challenge to stem the spread of COVID-19 is to track infected people with coronaviruses that can spread the disease. Although technological solutions such as contact traceability have been successful in some countries, they raise resistance in society due to privacy concerns [1]. The European Data Protection Board has published a guideline for governments to use this kind of technology, guaranteeing privacy and proper access to the data [2]. The solutions currently in consideration fall into two main groups: (1) personalized tracking of users from its geolocation and (2) individual monitoring of contacts. These groups correspond to the two waves of technological responses in Europe to the COVID-19 pandemic [3].

User tracking uses two sources of information: (i) data provided by telecommunication companies, which know precisely the position of the terminals, or (ii) the location provided by users of their own free will via GPS.

- advantages: precise information is available on the movements of people and their contacts, known or unknown, allowing the tracking and control of people;
- drawbacks: a threat to privacy.

With private tracking of contacts, every time two mobiles are nearby, they exchange an encrypted key via Bluetooth (BT). These keys remain stored in the device or at centralized servers.

- advantages: some privacy is maintained;
- drawbacks: the administration does not receive global data or statistics. Moreover, the information is available a posteriori, which makes prevention difficult (it prevents propagation, but not contagion).

Most governments have recommended using the second type of applications, advising against proposals based on individuals’ geolocation. Simko et al. [4] made a series of surveys over 100 participants to analyze their opinion about contact-tracking applications and privacy. It is a relevant study since the first part finished when some European countries were under different forms of lockdowns, and contact-tracing apps were not available yet. Between the first and the second study, several proposals appeared, such as the ones made by Apple and Google [5,6], the Massachusetts Institute of Technology (MIT) [7], the University of Washington (UW) [8], PEPP-PT [9], Inria [10], WeTrace [11], and DP3T [12]. The study shows that people are more comfortable using an existing mapping application that adds tracking for COVID-19 instead of using new apps, with reservations even if they provide ’perfect’ privacy. One of the main concerns is sharing data, preferring that Google or organizations such as the UN develop the application. In general, participants manifest a lack of trust in how their governments would use the citizens’ location data. They thought that it was unlikely that their government would erase the data after the crisis and also that they would use it for other purposes. Something in common for both studies was mixed feelings about using proximity tracking for the contacts and negative towards using any other data source.

Therefore, how can this be done while maintaining privacy? Alternatively, at least, allowing citizen controlling when and with who are they sharing the health data. This work proposes a third type: a process of dissemination in exchanging information with known and trusted contacts only.

- advantages: (i) privacy is maintained, (ii) the administration obtains aggregated information at the time, and (iii) the citizenry and the administration have the same data (transparency);
- drawbacks: the first exchange contains the data of only one node (in case someones hacks it and retrieves the information), and a critical mass is necessary.

**Figure 1:**
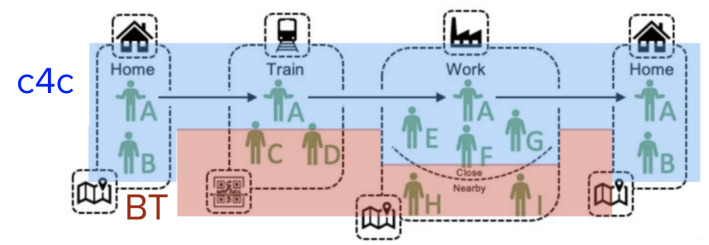
Integration of BT’s anonymous user tracking with a local broadcast application for close contacts (C4C). Image adapted from Ferreti et al. [13].

Throughout the day, we have contact with various people in different places: home, transport, work, or leisure. In each place we relate in different degrees (see Figure 1): closer (B,E,F,G) or farther (C,D,H,I). Private tracking of contacts via BT is useful for detecting risk in contact with unknown people and isolating those who have been in contact with the infectious agent to control the disease’s spread. The consensus process is useful to identify persons already infected or at risk in the immediate environment. A second potential is to provide aggregated statistical information to the health services [13].

Despite the efforts to develop technological solutions to track the propagation of COVID-19, the usage of the apps is not extended enough. That is why we propose an alternative solution. The consensus for COVID-19 (C4C) method is a variation of the consensus processes proposed by Olfati–Saber and Murray [14]. It is a dissemination process that allows a distributed calculation of the value of a function in a network, exchanging information only with direct neighbors without having global knowledge of the structure, size, values, or other characteristics of the graph. This process converges to a final single value for the calculated function. Privacy is maintained using this approach, the administration obtains aggregated information, and citizens and the administration have the same data, promoting transparency. One relevant limitation is that some critical mass is still necessary.

The rest of the paper is structured as follows. Section 2 reviews the usage of contact tracing apps developed worldwide. Section 3 explains how citizens can collaboratively create risk maps using a consensus process with their close contacts. Section 4 shows the results using La Gomera as an example: one of the Canary Islands, with 21,550 inhabitants. The Spanish government carried out a pilot project with its contact tracing application (RadarCOVID) on that island, so we decided to use the same scenario. Finally, Section 5 summarizes the main findings of this work.

## 2. Review of Contact Tracing Apps

**Figure 2:**
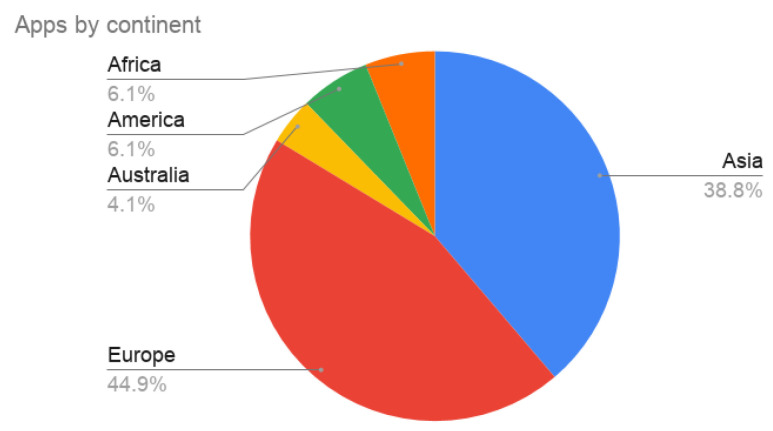
Distribution of the tracing apps by continent.

States have put their efforts into the development of mobile applications to track the spread of the SARS-Cov-2 over the population. The MIT Technology Review [15] has been collecting the different proposals. In the last checked update (28 February 2021), there are 49 registered apps. We have included in the studio the data from the application of the Spanish government, RadarCOVID (https://radarcovid.gob.es/, accessed on 17 May 2021), so we have analyzed 50 applications. Table 1 summarizes their characteristics. There are initiatives in the five continents, but most of the countries belong to Asia and Europe since they were the first places where COVID-19 appeared (Figure 2).

**Figure 3:**
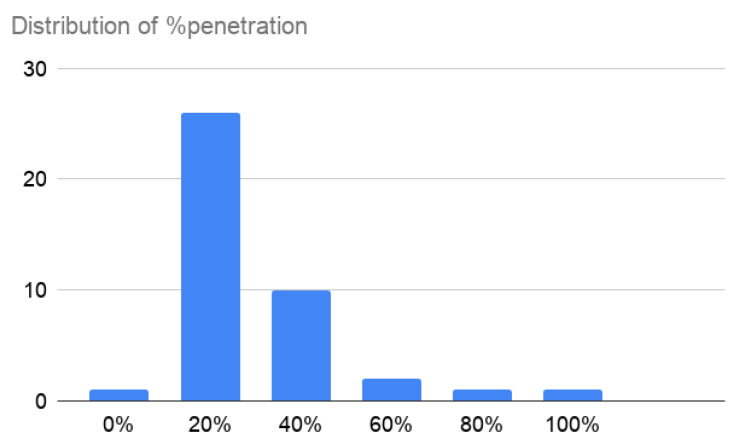
Distribution of the penetration of the app in the population.

The population that uses the applications varies from 9000 inhabitants in Cyprus or Gibraltar to 163,000,000 in India. The median value is 970,000. Regarding the penetration, the average value is 16.9%, ranging from 0.3% in cases such as Malaysia, Bangladesh, or Hungary, to 91% in Qatar. However, the data are not Gaussian (see Figure 3). The median is 12.3%, and the median absolute deviation 14.8%, which gives a fitter view of the actual penetration.

**Figure 4:**
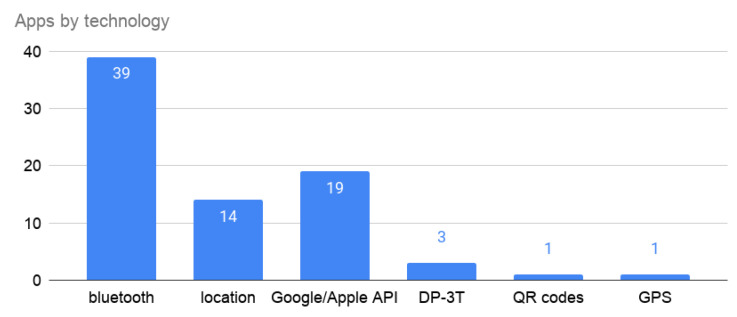
Technology used by the different proposals.

The Bluetooth technology is the solution that most countries have chosen (Figure 4), with 78% of the apps. Moreover, 38% of them use the Exposure Notification API provided by Google and Apple. Despite the recommendation to avoid location services, 28% of the apps use it.

**Figure 5:**
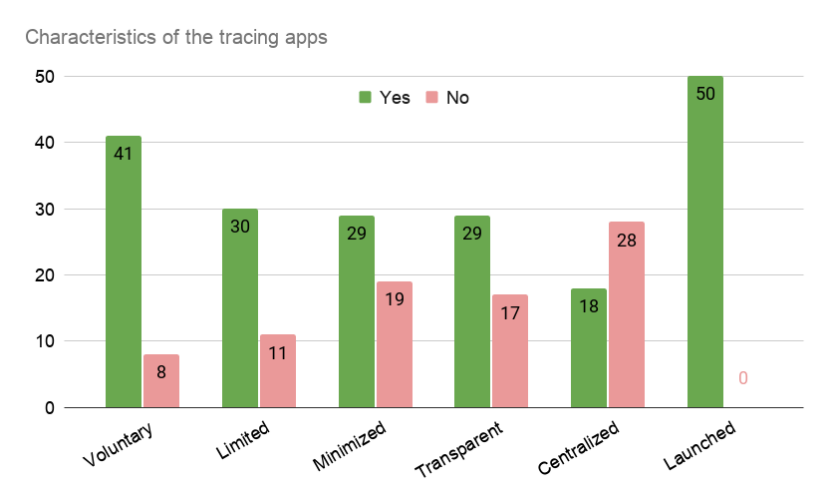
Characteristics related with privacy and the data treatment present in the available solutions.

Figure 5 shows that privacy concerns have been considered in general. The app considers five characteristics:

- voluntary: users opt-in to use the app or all citizens are compelled to download and use it;
- limitations: data have constraints to be used for purposes other than public health;
- destroyed: the data are automatically deleted in a reasonable amount of time or the the users can manually delete their data;
- minimized: the app collects only the information it needs;
- transparent: it has clear, publicly available policies and design, and open-source code base.

The voluntariness and limited information tracking are present in a proportion of 80–20. The remaining features: minimization of the information, transparency, and decentralization in a 60–40 balance.

In this situation, the usage of the apps is not extended enough despite the efforts to develop technological solutions to track the propagation of COVID-19. Figures show a total of 300 million installations or the available contact tracing applications, which means a 4.1% of the world population. Even considering that, in most cases, governments follow the recommendations to keep the citizens’ privacy at reasonable levels. For this reason, we propose a third method that can work with the penetration obtained with current applications.

## 3. Collaborative Risk Map Generation

The consensus for COVID-19 (C4C) proposal works over a contact network with non-reciprocal relationships. C4C does not model the propagation of COVID-19. It aims to use the acquittance network of the people to exchange the risk index, so the C4C is about information propagation, no virus propagation. Therefore, we can choose to have undirected (symmetric) or directed links.

If we use undirected links, a well-connected person can receive hundreds of requests. Many of them will not be close contacts from his or her point of view. Our model allows users to choose a small number of close contacts (maximum 15) who receive their health information (the risk index) to maintain privacy. The limitation of output relations avoids the existence of hubs just for the out-degree. The in-degree has no limits. Therefore, the underlying structure is a directed graph. The original consensus algorithm works over non-directed graphs, so we have to extend the model to consider this case (see Section 3.2).

Let G=(V,E) be a non-directed network formed by a set of vertices *V* and a set of links E⊆V×V where (i,j)∈E if there is a link between the nodes *i* and *j*. We denote by $N_{i}$ the set formed by the neighbors of *i*. A vector $x={\left(x_{1},\ldots ,x_{n}\right)}^{T}$ contains the initial values of the variables associated with each node. Olfati–Saber and Murray [14] propose an algorithm whose iterative application converges to the mean value of *x*.

(1) $$x_{i}\left(t+1\right)=x_{i}\left(t\right)+\varepsilon \sum_{j\in N_{i}}\left[x_{j}\left(t\right)-x_{i}\left(t\right)\right]$$

The authors demonstrated that this consensus process converges to the average of the initial values when $\varepsilon <\frac{1}{\max d_{i}}$, being $d_{i}$ the degree of node *i*. There is an equivalent matricial formulation.

(2) $$x\left(t+1\right)=\underset{P}{\underset{︸}{\left(I-\varepsilon L\right)}}x\left(t\right),\;\mathrm{with}\;L=D_{A_{G}}-A_{G}$$

where *I* denotes the identity matrix, and *L* is the Laplacian of *G*, calculated from the degree matrix $D_{A_{G}}$ and the adjacency matrix of the graph $A_{G}$. *P* is called the Perron–Frobenius matrix and governs the consensus process’s collective dynamics.

### 3.1. Consensus as a Distributed Counting Mechanism

Without losing generality, we can extend the consensus process over *m* independent variables, just considering $x_{i}=\left(x_{i}^{1},\ldots ,x_{i}^{m}\right)\in {\mathbb{R}}^{m}$. The consensus process over these vectors is equivalent to *m* independent consensus process, each one over each component. The complete process converges to the average of the initial components.

By adding one additional element $y_{i}\in \mathbb{R}$, we can determine the size of the network at the same time. In each node, the state is defined by an extended vector

(3) $$\left(x_{i}|y_{i}\right)=\left(x_{i}^{1},\ldots ,x_{i}^{m}\;|\;y_{i}\right)$$

and, initially, $y_{i}=0\;\forall i$. Without losing generality, we can introduce an additional node in the network whose initial values are

(4) $$\left(x_{0}|y_{0}\right)=\left(\underset{m}{\underset{︸}{0,\ldots ,0}}|\;1\right)$$

This node does not affect the result since it does modify the values of any component of $x_{i}$. When the consensus process converges, each node calculates how many participants have chosen each option dividing the final vector $x_{i}$ by the value obtained in the last column $y_{i}$ to determine the overall result, as shown in Equation (5).

(5) $$\begin{array}{cc} \frac{x_{i}\left(t\right)}{y_{i}\left(t\right)} & =\frac{\left(\langle x^{1}\rangle ,\ldots ,\langle x^{m}\rangle \right)}{1/n}=\left(n\langle x^{1}\rangle ,\ldots ,n\langle x^{m}\rangle \right) \end{array}$$

(6) $$\begin{array}{cc} \; & =(\sum_{j}x_{j}^{1},\ldots ,\sum_{j}x_{j}^{m}) \end{array}$$

where n=|V|.

Table 2 shows an example of the initialization of a network with six nodes. The nodes choose between 3 options, being the choice of each one x(0)=(1,2,1,3,3,1). Therefore, $\left(x_{1}|y_{1}\right)=\left(1,0,0|0\right)$, $\left(x_{2}|y_{2}\right)=\left(0,1,0|0\right)$, $\left(x_{3}|y_{3}\right)=\left(1,0,0|0\right)$, and so on.

**Figure 6:**
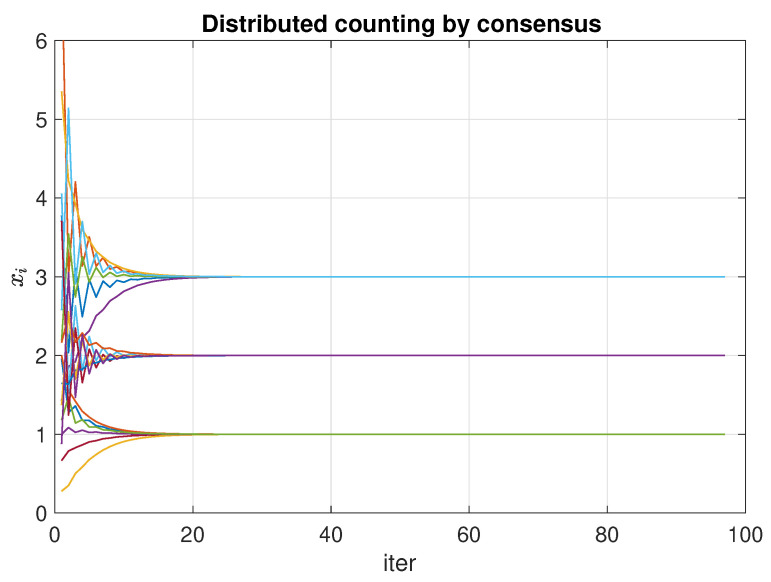
Evolution of the consensus process for calculating the result of a distributed count. Random network with n=6 nodes and m=3 options. The options are x(0)=(1,2,1,3,3,1), which gives a result of (3,1,2).

We can see in Figure 6 the evolution of the consensus process over each one of the components of the vector $x_{i}$. The network converges to the total result $x_{i}\left(t\right)=\left(1/2,1/6,1/3\;|\;1/6\right)$, and applying Equation (5) each node obtains the total counting

(7) $$x_{i}\left(t\right)=\left(\frac{1/2}{1/6},\frac{1/6}{1/6},\frac{1/3}{1/6}\right)=\left(3,1,2\right)$$

### 3.2. Consensus on Directed Networks

The algorithm of Olfati–Saber and Murray needs the Perron matrix *P* to be double stochastic for the consensus to work. In a double stochastic matrix, all the rows and all the columns sum one. That means that the network’s adjacency matrix has to be symmetric and, therefore, the graph has to be undirected. As C4C is based on non-reciprocal relations, the underlying graph is directed, and the associated Perron matrix will be stochastic by rows only. In this situation, the equations of the consensus model converge, but to a value different from the average of the initial values.

**Figure 7:**
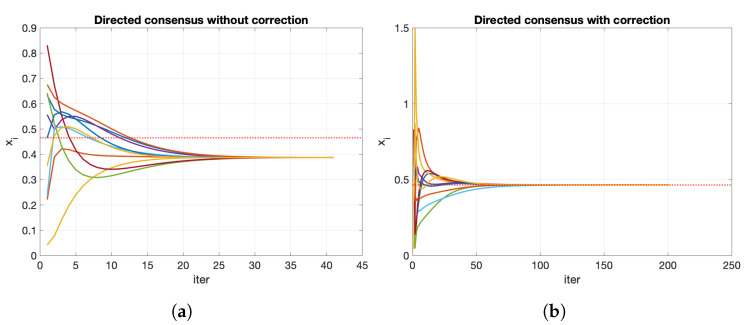
Consensus process over directed networks: (**a**) without the proposed correction, the network converges to a common value 〈x〉=0.3876 different from the initial mean; (**b**) with the proposed correction, the network converges to the average value at 〈x〉=0.4660=〈x(0)〉.

Figure 7a depicts the evolution of the consensus process over a directed network using the general formulation. The network converges to a common value (0.3876), which is different from the mean of the initial values (0.4660). Therefore, we need some mechanism to correct this deviation.

To explain the adaption of the formulation, we use the matricial representation of the consensus process (Equation (2)). Inspired in the Dominguez-Garcia and Hadjicostis matrix scaling algorithm [16], we define an iterative process to convert the Perron matrix into a double stochastic one. This modification consists of including the consensus process in the master equation of the consensus process instead of using a general one. The process begins with a row stochastic matrix $\bar{P}$

(8) $$\bar{P}=\left(I-\Delta \left(0\right)*L\right)$$

which is a local version of the Perron matrix, defined using each node’s degree Δ(0) instead of a common ε value for all the nodes (see line 4). In each iteration, the matrix is scaled following the expression

(9) $$P\left(t\right)=\bar{P}\Delta \left(t\right)+\left[I-\Delta \left(t\right)\right]$$

where $\bar{P}$ is a local Perron matrix and Δ(t) is updated as Algorithm 1 describes. The scaling algorithm is an iterative process that executes until there are no more changes in the *P* matrix. We do not have to wait until we obtain *P* to execute the consensus process. We can combine the matrix’s scaling (line 11) with the consensus value calculation in the same step (line 12). With the correction of the proposed algorithm, the process converges to the mean of the initial values (Figure 7b).
**Algorithm 1** Matrix scaling and consensus (collective)1:init x(0)2:$L=D_{A}^{out}-A$3:$\Delta \left(0\right)=D_{L}^{-1}$4:$\bar{P}=\left(I-\Delta \left(0\right)*L\right)$5:$P\left(0\right)=\bar{P}\Delta \left(0\right)+\left[I-\Delta \left(0\right)\right]$6:π(0)=0,η(0)=17:**repeat**8: π(t+1)=P(0)π(t)9: $\eta \left(t+1\right)=\max\left(\pi (t),\max\eta (t)A\right)$10: $\Delta \left(t+1\right)=\Delta \left(0\right)\frac{\pi (t+1)}{\eta (t+1)}$11:  $P\left(t+1\right)=\bar{P}\Delta \left(t+1\right)+\left[I-\Delta \left(t+1\right)\right]$12:  x(t+1)=P(t+1)x(t)13:**until**x(t) converges

The adaptions of the scaling algorithm proposed by Dominguez-Garcia and Hadjicostis to the equations of the consensus process implies:

1. the calculation of Δ(t), in lines 3 and 10;
2. the definition of the matrix $\bar{P}$, as a local variation of the Perron matrix, in line 4;
3. how π(t) updates, in line 8.

### 3.3. Map Generation

Once we have defined the algorithm for consensus processes over directed networks, the goal is to create a citizen network in an area (town, province, state, or any other administrative division) that uses it to make a risk map collaboratively.

We can describe the process as follows:

- the nodes of the network are the population: the inhabitants of the city;
- the map (a town, an island or any other region) is divided into *m* zones. For instance, postal codes in a city;
- each node *i* has a vector $x_{i}=\left(x_{i}^{1},\ldots ,x_{i}^{m}\right)$ with as many components as zones, that is, *m* components;
- using the algorithm of Section 3.1, all inhabitants calculate the same vector that aggregates the information of the zones. For instance, sum the values for each postal codes;
- we use a directed network, so the algorithm used is the variation of Section 3.2.

**Figure 8:**
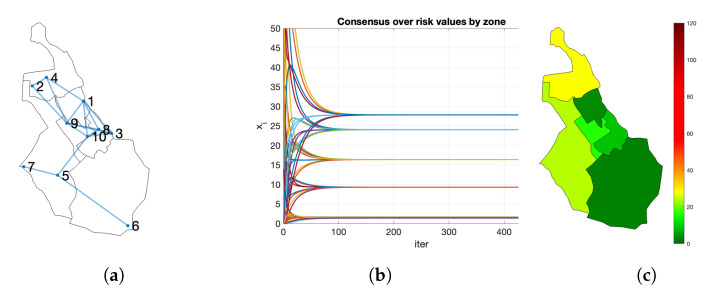
Example of creation of a risk map in a city: (**a**) network of citizens in their living zone; (**b**) consensus process; (**c**) risk maps values obtained by the process.

Figure 8 shows an example for the creation of a risk map. Let us consider a network of citizens with n=10 nodes and a city divided into m=6 zones. The initial risk values are (3,2,10,54,30,3,18,8,5,28) and the zones corresponding to each node z=(3,1,5,1,2,6,2,5,4,4). Node i=1 has a risk value of $r_{1}=3$ and she lives in zone $z_{1}=3$, so $x_{1}\left(0\right)=\left(0,0,3,0,0,0\right)$. For the rest, $x_{2}\left(0\right)=\left(2,0,0,0,0,0\right)$, $x_{3}\left(0\right)=\left(0,0,0,0,10,0\right)$, and so on. When the consensus converges, all the nodes have the same copy of the vector, $x_{i}\left(t\right)=\left(28,24,3,16,9,2\right)$. Observe that it matches with the mean values for each zone. Zone 1 is the one with a higher risk.

(10) $$r_{1}=\frac{x_{1}\left(0\right)+x_{4}\left(0\right)}{2}=\frac{2+54}{2}=28$$

We have chosen the census districts from the National Institute of Statistics (INE) of Spain. The sizes of the districts are relatively homogeneous, having between 900 and 3000 inhabitants each. It is easily scalable, aggregating the information in bigger administrative units. Moreover, they never provide statistics with less than 100 persons to avoid reidentification.

Inhabitants share a risk index (RI) that measures their probability of being infected by COVID-19. The risk in a census district depends on the RI of all the people that live in it. The RI could integrate data from different sources: medical symptoms, symptoms of close contacts, age, family situation, or habitability conditions. In this work, we use the same measure as the emergency service 112 (https://coronavirus.comunidad.madrid/, accessed on 17 May 2021). The risk value depends on medical symptoms: shortness of breath (60 points), fever (15), coughing (15), or close contact (29). Over 30 points, it is considered that the person has been infected.

We use the following notation:

- $r_{i}$: risk index of node i,i=1,…,n.
- $\left(x_{i}|y_{i}\right)=\left(x_{i}^{1}\ldots ,x_{i}^{m}\;|\;y_{i}\right)$: vector with the risk map values in node *i*.
- $R_{i}=\left(r_{i}^{1},\ldots ,r_{i}^{m}\right)$: complete risk map calculated in node *i*.

Let us assume an extra node representing an administrative unit, such as the town hall, acts as the $x_{0}$ node (see Section 3.1). Algorithm 2 describes the complete process.
**Algorithm 2** Risk map creation1:calculate $r_{i}$2:init $\left(x_{i}\left(0\right)|y_{i}\left(0\right)\right)=\left(0,\ldots ,r_{i},\ldots ,0\;|\;0\right)$,3:execute Algorithm 1 until convergence4:calculate $R_{i}=\frac{x_{i}\left(t\right)}{y_{i}\left(t\right)}$

Some important remarks related to the process are:

1. the position of $r_{i}$ in $x_{i}\left(0\right)$ corresponds to its census district;
2. each node executes a local version of Algorithm 1;
3. the first exchange is the only moment in which vectors contain individual values: the risk and the census district of *i*. We assume that there are no privacy concerns since the node would share this information with its $N_{i}$ trusted neighbors;
4. in the following exchanges, the received vectors $x_{j}\left(t\right)$ contains aggregated information. As the neighbors of *j* remain unknown for *i*, it is complicated to track back the data.

It is a successive refinement mechanism: there is a map available at any time, and the longer the algorithm executes, the fitter the risk values are. The final risk map *R* is equal to the one obtained by a centralized process with all the risk indexes available. You can find an example in Section 4.2.

## 4. Results

The purpose of this section is to validate the effect of an app with the characteristics of C4C, based on the algorithms presented in Section 3, for the COVID-19 propagation. Such a tool would provide the citizens the risk map of their town. With this information, they can decide if they modify aspects of their daily routines.

As there is no other tool with similar characteristics, we will try to simulate its effects in the propagation of COVID-19 and compare it with the impact of common contact tracing apps. To compare the results, we have chosen RadarCOVID: the contact tracing application created by the Spanish government.

We have run several simulations using the same scenario as RadarCOVID. The authors of the application have published the study’s conclusions, but it contains the results about the application usage [17]. Therefore, for this scenario, we need to recreate an epidemic model for SARS-Cov-2 and the mobility patterns of the people (Section 4.1). To recreate the network’s structure generated for a hypothetical C4C application, Section 4.2 proposes a distribution following how people could be connected in small towns. Once the topologies of the networks and the contagion process have been established, the rest of the section shows the results of the contact tracing application (Section 4.3), the behavior of the population using information from risk maps (Section 4.4), and the combination of both methods (Section 4.5).

### 4.1. Population and Infection Model

As an application example, we have chosen La Gomera: one of the Canary Islands, with 21,550 inhabitants. The National Institute of Statistics divides the island into 14 census districts. We need a model of the population of the area and how they move to incorporate it into an epidemiological model to simulate the propagation of SARS-Cov-2. Due to privacy restrictions, the 14 districts are grouped into two, called Valle Gran Rey and San Sebastian de La Gomera. The only available information is the aggregated number of movements from and to each one on these two areas. Nevertheless, the aim was not to have a detailed model of the movements of the individual citizens, but something coherent with the observations and the actual public data available.

We make the following assumptions

1. Lévy flights model properly the commuting movements of the people;
2. any person has the same probability to get infected;
3. the propagation of SARS-Cov-2 follows an SEIR model.

**Figure 9:**
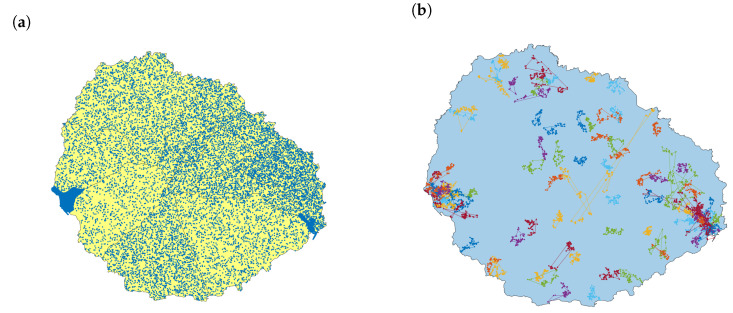
Population model in La Gomera: (**a**) Population distribution; (**b**) simulated commuting movements sample with 100 paths using Lévy flights.

The population that lives in each area is publicly available through the Spanish National Statistics Institute (INE). The network has as many nodes as inhabitants. For each node, we generate the coordinates for their home address at random. The data available are just the number of residents in each census district. In addition to the two biggest towns on the island, Valle Gran Rey and San Sebastian de La Gomera, many tiny villages and individual houses spread along all the island. The two first towns are included in a district for themselves (Figure 9a shows clearly both by their density). We have considered a uniform distribution in the absence of additional data.

The people’s movements along the day are simulated using recurrent Lévy flights [18]. Lévy flights are random walks where the step-length follows a Lévy distribution. This type of search involves mostly short move steps combined with sporadic longer move steps. Each person has assigned a path with 96 points (taken every 5 min during 8 h) that begins and ends at his or her home location (Figure 9b). With this approach, we model the commuting trips when people go to work, study, or other regular activities. The recurrent model includes the return to the origin, which is typical in daily human movements. The model parameters are the same as proposed in the referred paper: the distribution of the displacements follows a power law of parameter variable for each node β=1.75±0.15, scaled to the size of the island with σ=100 as factor. The Matlab library uses the McCulloch’s implementation [19] to generate Lévy flights with these two parameters β and σ.) Trips avoid going out of the bounds of the island. Two consecutive points at a distance lower than 10 m are unified.

We have validated the model by comparing the movements with the data available in the study on mobility based on mobile phones carried out by the Spanish National Statistics Institute (INE) in 2020 (https://www.ine.es/en/experimental/movilidad/experimental_em_en.htm, accessed on 15 January 2021). In this study, La Gomera was divided into two areas to avoid reidentification and keep privacy: Valle Gran Rey (VGR), with 10,393 inhabitants, and San Sebastián de La Gomera (SSG), with a population of 11,110. The average daily mobility from March to June was 429±148 persons for VGR and 523±221 for SSG. The simulation with Lévy flights throws a total flow of 464 persons for VGR and 668 in the case of SSG. Therefore, we can consider that the movements are in the same magnitude order, so we assume that Lévy flight model is coherent with actual commuting trips.

To simulate the close contacts, we use the same criteria as the contact tracing app [17]: a close contact is defined when two persons are at 5 m at most and during 15 min since with 2 m it only obtains a 78% accuracy. The distance is estimated from the strength of the Bluetooth signal. Initially, a range between 63 dB and 74 dB was considered, but after a few days, it was recalibrated, and the range [53 dB, 74 dB] was established. The result is a daily risky contact matrix of dimension 21,550 × 21,550.

To simulate the spread of the COVID-19, we use an SEIR model, where each person can be in one of these states:

- Susceptible (S): a person that has not the disease and can be infected;
- Exposed (E): someone that has been in contact with an infected person. He or she cannot infect others yet;
- Infected (I): someone with symptoms that can infect other people;
- Recovered (R): people cured that are immune to the disease.

Its parameters follow the literature’s findings that have analyzed the COVID-19 propagation [20]. Particularly, the incubation time is 7 days, so β=1/7, the probability of infection σ=0.1 and the recovery time is 15 days, so γ=1/15. Another relevant parameter is the time that elapses from the appearance of the first symptoms until the person receives a positive diagnosis. We use an optimistic period of 48 h. The purpose of the model is not to predict precisely the behavior of the disease. Therefore, the model provides the consensus process with different scenarios to check the risk maps’ accuracy.

**Figure 10:**
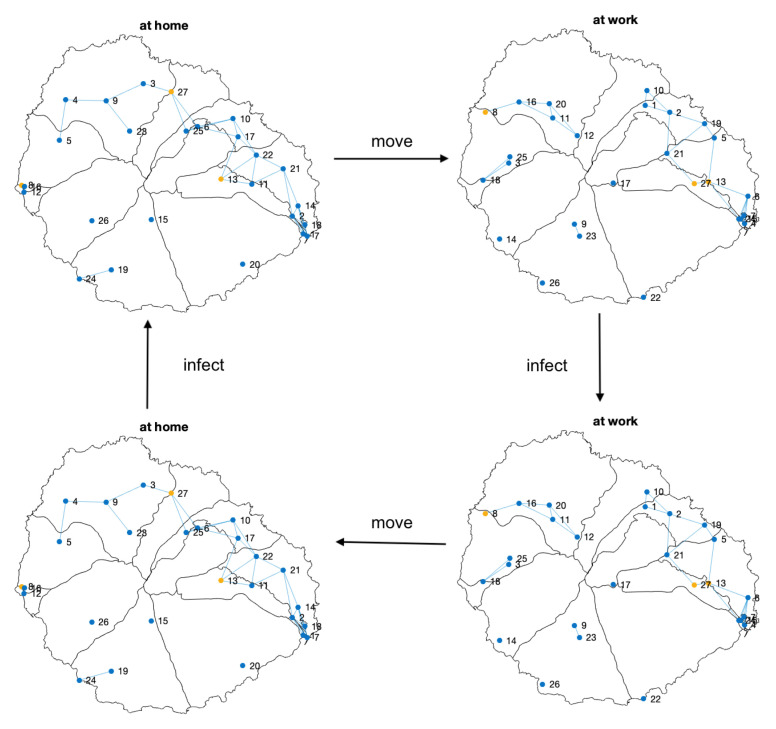
Scheme of the stages involved in the two-step contagion model considered. The population moves alternatively between the home and the working location. Carriers can infect other people in both places. The cycle consists of a sequence movement → infection → movement → infection. Only some nodes are shown. Color nodes indicate the health state of the corresponding person: susceptible (blue), exposed (yellow), infected (red), recovered (purple). Two nodes are connected if they are close enough to transmit the COVID-19.

Infection models usually assume a single infection stage per day. Nevertheless, the domestic environment has demonstrated to be a significant source of contagion [21,22]. For that reason, we have defined a two-step model, similar to the model proposed by Gomez, Soriano, and Arenas [23], but including a second infectious phase. In Figure 10, we present the general scheme of the infection processes considered in our model, that, considering that people start at their home location, can be summarized as follow:

1. People start at their home location;
2. They move along the day, interacting with the other persons;
3. Nodes update their state according to the epidemic model and the contact matrix;
4. They go back to their home locations;
5. A new infection stage is performed at home.

Once the update is completed, a new cycle begins.

The results of the model show that there have been a total of 37,900 risky daily contacts, i.e., contacts of more than 15 min at less than five meters. That is the result considering the total population of La Gomera. If we consider only the 3000 people who have the contact tracing app installed, this number remains at 864 daily contacts, which would be the maximum number of alerts that the application would launch. Our results can be compared with the observations of the pilot project that evaluated the use of RadarCOVID contact tracing app [17]. The actual number of alerts triggered by the app was 821. On average, each user has 6.38 contacts, in line with the 6.4 identified in the pilot project. Supercontagiators appear, with more than 30 contacts. This would also indicate that the model is not far-fetched since it is observed in different outbreaks. We can assume that the infection model reflects the observations in the pilot project, so the assumptions about the parameters of the SEIR model and the contact matrix generated by the Levy flights and the two-step contagion process are coherent with the observations.

**Figure 11:**
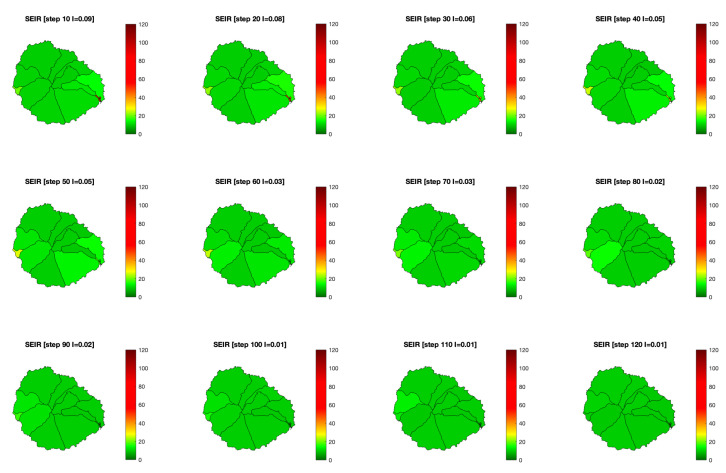
Evolution of the risk map with an SEIR model in La Gomera island, without considering any app. Color bar indicates the value of infected population (I).

With all this data, we can now see how a COVID-19 infection would have behaved on the island and the effect RadarCOVID would have had over the base scenario (Figure 11). In the pilot project, they started with 100 infected users on 10th July, and then they added three waves of 50 to 100 more simulated infections on July 13th, 15th, and 17th. In the model, 300 randomly infected people have instead been introduced across the island at instant zero. Of these, about 50 had the tracking application installed. The result is similar, with about 400 infected people being added to the network corresponding to the week of July 10–17.

### 4.2. Risk Map Creation

The consensus process described in Algorithm 2 obtains the actual risk map if all the inhabitants participating in the process. However, as we have seen the results with contact tracing apps in Section 4.1, this is a utopic scenario. Therefore, we assume that

1. only a subset of the population participates in the creation of risk maps;
2. people has an small subset of confident contacts among family, friends, and colleagues from work or studies;
3. risk values are uniformly distributed among all the population.

With these assumptions, let us check if the risk maps created by a reduced subset of the population reflect the reality with enough accuracy.

Usually, the contact network of a person is modeled with an egocentric exponential random graph, which has been used in other works in the COVID-19 context [24]. Nevertheless, the size of the island produces exponential decay due to the finite size effect of such a small population. In these cases, a Weibull distribution fits better with the data [25,26].

**Figure 12:**
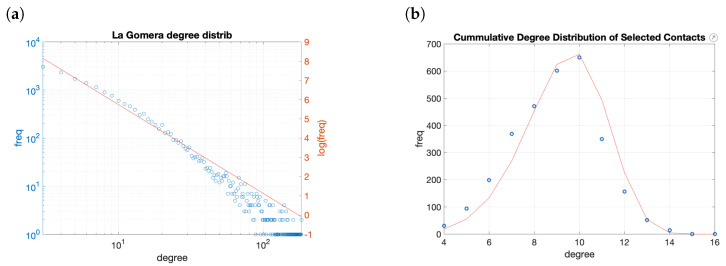
Cumulative degree distribution of the networks: (**a**) Complete contact network with a power-law with α=−1.7; (**b**) random selection of contacts. It follows a Weibull distribution with parameters α=13.4, λ=0.48.

As in the case of the commuting trips, we have no direct data about the actual structure of the relationships in La Gomera. To simulate it, we have used previous works in which towns between 4000 and 70,000 were analyzed. We have assumed that the structure of the citizens’ relationships in Twitter is a reflex of the actual relations among all the people. We have analyzed the network formed by the Twitter account followers of the town halls of cities of similar sizes [27]. The cumulated degree distribution, in this case, follows a power law with parameter α≈−1.7. Therefore, to model the entire population’s contact network, we have generated a preferential attachment network following the same distribution (Figure 12a). This generates a network of 58,000 contacts and a maximum degree of 876.

As a potential application would bound the number of closer contacts, we choose a subset of the potential links. A reasonable limit is 15 contacts, five of each type (family, colleagues, and friends). If we choose randomly 9±2 of the contacts, the resulting network has 26,500 links and the number of connections vary from 4 to 16, with a Weibull degree distribution with scale parameter α=13.4, and shape character λ=0.48 (Figure 12b). The Lilliefors test rejects the null hypothesis for the normality of the data and gives a *p*-value = 0.5132 to follow a Weibull distribution. The strategy used to generate the contact network for C4C matches with the findings in the literature for similar networks.

Just 3000 persons have participated in the RadarCOVID contact tracing app. To compare the results, we will use a graph of the same size. Therefore, we have created a network with 3000 nodes and mean degree 10, varying from 4 to 16, generated as a subset of social network contacts. Over this scenario, the inhabitants can create their town’s risk map at the end of the day. We assume that no additional measures, such as social distancing or limitations of movements, are taken.

**Figure 13:**
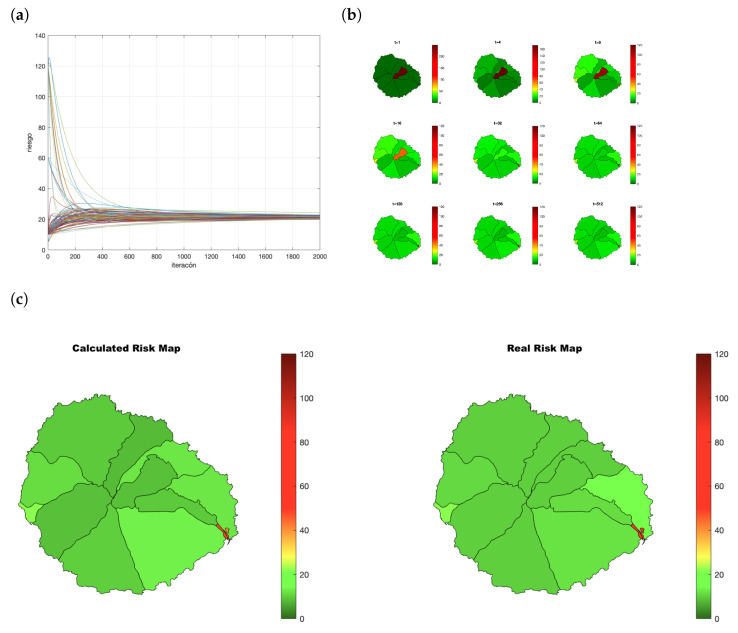
Evolution of the consensus in the creation of a risk map. (**a**) Convergence of the process; (**b**) evolution of the map build by one random node; (**c**) map created by consensus versus real risk map.

As an example, let us consider the situation after 30 days. Each node has a vector of 14 components, one for each census district $R_{i}\left(t\right)$$=(x_{i}^{1},\ldots ,x_{i}^{14}\;|\;0)$. Let $r_{i}$ be the risk index of *i* and *k* the census district in which *i* lives. The first value $R_{i}\left(0\right)$ starts with all components $x_{i}^{l}=0,\;l\neq k$ and for the census district of the node $x_{i}^{k}=ri_{i}$. Each node executes the process detailed in Algorithm 2. The evolution appears in Figure 13a and the resulting maps in Figure 13b. When the consensus process converges, the values obtained by the node are $R_{i}\left(t\right)$ (see Table 3). The 3000 inhabitants have obtained similar values. *R* are the real risk values for each census district considering all the population. In this example, the districts with higher risk (7, 8, 9, and 11) are properly identified by the participants (Figure 13c).

To sum up, a contact network formed by between 4 and 15 close contacts allows the creation of a significant risk map compared with the real risk of the census districts. The modifications described in Section 3 produce the expected result even with a subset of the complete population.

In the remaining sections, we analyze the effect contact tracing and risk maps on the propagation of SARS-Cov-2, separately and combined.

### 4.3. Evolution with Contact Tracing App Active

The first analysis tries to determine if the problem with tracking applications is that they need a large percentage of the population to use them. Some works suggest that tracking applications need at least 60% of penetration to be effective [28]. We have simulated the propagation of COVID-19 in three scenarios:

- worst case (blue): no measures taken;
- best case (red): all infected detected and isolated;
- isolation of traced users (yellow): people with contact tracing app isolated 48 h after the first symptom appears, as well as all other persons that have been in contact with them.

**Figure 14:**
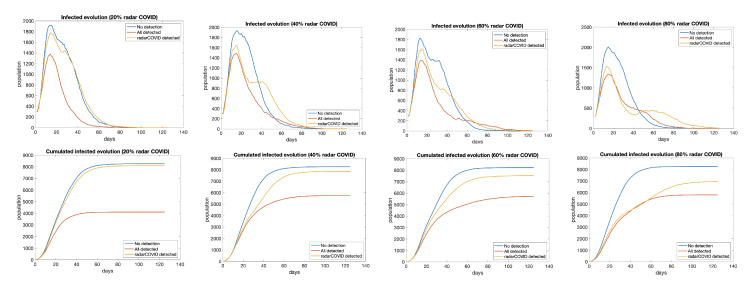
Evolution of infected in three scenarios: no isolation (blue), total isolation (red), and isolation for traced users (yellow), from 20% to 80% of users. (**top**) Total infected by day and (**bottom**) cumulative infections.

To evaluate the impact of the penetration of the app, we have considered 20%, 40%, 60%, and 80% of the total population using the app (Figure 14). The effect of contact tracing apps in the propagation is almost irrelevant with 20% of users. There are just little differences between 40% and 60%, so we don’t need to arrive at the 60% of penetration since we have shown that 40% of active users would be enough. With 80%, the transmission is almost controlled.

### 4.4. Effect of Risk Maps in Infections

**Figure 15:**
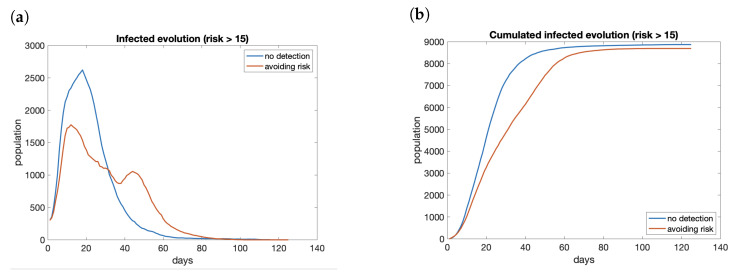
Evolution of infected with and without considering the risk map: (**a**) evolution; (**b**) cumulated.

The effect of having a risk map available and avoid areas with high risk does not reduce the total number of infections significantly. It reduces the peak (see Figure 15a), but it keeps the propagation active for more days and the total number of cases at the end of the period is similar (Figure 15b). Therefore, we can conclude that the use of a risk map does not reduce the number of infected people in the long run. Nevertheless, the effect is a reduction in the peak, smoothing the curve of infections. The main benefit of this situation is to avoid the collapse of health services if the cases are reduced enough. We do not have available the information to check that hypothesis.

Since neither the isolated use of a contact tracing application nor the availability of a risk map seems to be enough to control the transmission, we have combined both tools and simulated the results.

### 4.5. Contact Tracing and Risk Maps Combined

Finally, we have tested the combination of risk maps and contact tracing. The behavior of the people follows these rules:

1. people that live in an area with medium or high risk do not go out from it;
2. people that live in a low-risk area move freely but do not enter risky ones;
3. tracing app notifies exposure in 48 h. People that receive a warning from the application isolate themselves and stay at home.

The risk denomination as low, medium, and high follows the same criteria defined for the individual risk index specified in Section 3.3. We consider high risk a value >30 since symptoms appear in the population, medium risk between 15 and 30, and low risk any region under 15.

**Figure 16:**
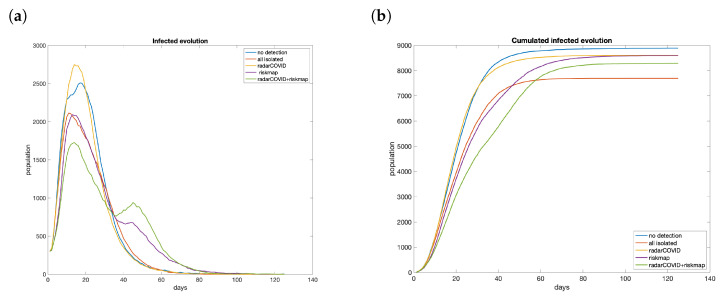
Evolution if the number of infections with different technological solutions was applied with 15% of penetration: (**a**) evolution; (**b**) cumulated.

Five scenarios are analyzed: no isolation, total isolation, limited movement by risk map, isolation by contact tracing, and risk map plus contact tracing. Considering the case with 3000 users (15% of the population of La Gomera), we see that contact tracing or risk maps have a low effect on their own in controlling the propagation (Figure 16a). The total number of cases without constraints reach 8890 persons. RadarCOVID barely reduces the contagion, with 8605 infected. On the other hand, the only effect of including risk maps is to smooth the curve of infections, reaching 8594 infected people. However, with both strategies combined, we obtain a significant reduction in total infected, until 8290, reducing 50% the difference with the best case, in which 7695 persons were infected (Figure 16b). Furthermore, in all cases, the peak is reduced, which can be translated to a lower number of hospitalizations. This could help health authorities manage the situation and take more efficient measures once the saturation has been avoided.

## 5. Conclusions

Technology can be an essential ally to control the transmission of COVID-19. Nevertheless, concerns about privacy and the possible use of the data after the pandemic have made difficult the implantation of technological solutions.

This work proposes an alternative for users to create risk maps collaboratively, called C4C. This approach executes a consensus process that uses local information and data from the direct neighbors to calculate the value of a shared function. In our case, the values are the risk index of the different districts that form the town. Close contacts (family, colleagues, and friends) define the network relationships, whom we warned about being infected. The data exchanged are an aggregation, and it is not possible to reidentify the personal information. At the end of the process, all the participants obtain the same copy of the complete risk map. However, the effect of constraining the movements using the information on risk maps reduces the peak and smoothens the evolution of the infection.

Current technological solutions are based on contact tracing. Many countries have implemented their own applications to alert when a person has been in contact with someone infected after being diagnosed with COVID-19. The Spanish government conducted a pilot study. We have followed the same conditions to validate the models built for the experiments. After validating the network structure and the mobility pattern, we have simulated the propagation of SARS-CoV-2 in different scenarios. From the experiments, we can conclude that:

- A relatively high percentage of users is necessary for it to be effective. If we consider that approximately 70% of the population has a smartphone, we would be talking about the need to install it on 55% of the devices, something like 19 million users—an unattainable figure if we look at the percentages that have been achieved in the countries around us. It is necessary to combine it with other tools so that a lower rate is sufficient.
- Availability of risk maps reduces the peak of infections, but not the total number. Despite its benefits to control the saturation of the health system, it is not enough to stop the transmission.
- The combination of the information from risk maps to avoid areas with a high index of infections and alerts of exposure obtain good results even with relatively low penetration of the apps.

In addition to these conclusions from the results of the experiments, there are some other considerations to take into account.

- A high number of users implies an increased number of alerts. The health system must be prepared to deal with them and test all suspicious persons.
- tracking applications are not a substitute for professionals but a supplement. We do not need contact tracing apps to alert about cases in the family, work colleagues, or friends. It is beneficial to identify possible contacts with strangers: on public transport, in stores, or entertainment venues.
- This type of application is vital in the early stages of the epidemic but is overflowing when there is community transmission.
- Contact tracings are safe applications, but that does not exempt us from privacy threats by social engineering mechanisms, such as the scenarios that appear in Anonymous tracing, a dangerous oxymoron, or also by the platform itself, as is the case of Google Play Services, as indicated in the report ’Contact Tracing App Privacy: Europe’s GAEN Contact Tracing Apps shares what Data.’ So do not be confident and take the same precautions as with other apps.

Once we have checked the theoretical validity of the proposal, we pretend to create a prototype of the distributed application as future work. The deployment of such a solution introduces additional problems to be addressed when actual users install and activate the application. Most of them involve technical details, such as the platform to allow truly distributed communications, security, robustness under deliberate attacks, and fake data, or synchronization among users when they activate the application at different moments.

The usage of agent-based models (ABM) allows considering individual behaviors. In this work, all agents behave equally and fulfill all the recommendations from health authorities. As an extension to the current model, we plan to include, for instance, people with different risk perceptions, different isolation measures, or segregate people by age. The consideration of these improvements generates more accurate models to make better-informed decisions.

## Figures and Tables

**Table 1:** Tracing apps in the world. **Dep**: deployer, government (gov), private (priv), academic (acad), and NGO, **Pen**: penetration, **Tech**: used technology, **Dest**: destroys data when finished, **Min**: minimized data, **Transp**: transparency, **Central**: centralized or decentralized data storage, **Status**: launched or in development (Source: Adapted from MIT Technology Review [15]).

Name	Location	Continent	Dep	Users	Pen	Tech	Voluntary	Limited		Min	Transp	Central	Status
Algeria’s App	Algeria	Africa											Launched
COVIDSafe	Australia	Australia	gov	7,160,909	28.64%	Bluetooth	Y	Y	Y	Y	Y	Centralized	Launched
Stopp Corona	Austria	Europe	ong	600,000	6.77%	Bluetooth, Google/Apple	Y	Y	Y	Y	Y	Decentralized	Launched
BeAware	Bahrain	Asia	gov	400,000	25.49%	Bluetooth, Location	Y	Y		N	N	Centralized	Launched
Corona Tracer BD	Bangladesh	Asia	priv	500,000	0.3%	Bluetooth, GPS	Y	Y	N	N		Centralized	Launched
Coronalert	Belgium	Europe	gov	970,000	8.37%	Bluetooth, Google/Apple, DP3T	Y	Y	Y	Y	Y	Decentralized	Launched
ViruSafe	Bulgaria	Europe	priv, gov	55,000	0.79%	Location	Y	Y	Y	N	Y	Centralized	Launched
COVID Alert	Canada	America	priv	5,314,026	14.03%	Bluetooth, Google/Apple	Y	Y	Y	Y	Y	Decentralized	Launched
Chinese health code system	China	Asia	gov			Location, Data mining	N	N	N	N	N	Centralized	Launched
CovTracer	Cyprus	Europe	acad	9000	0.77%	Location	Y	N	Y	Y	Y	Decentralized	Launched
eRouska	Czech Republic	Europe	gov	240,000	2.25%	Bluetooth	Y	Y	Y	Y	Y	Decentralized	Launched
Smitte|stop	Denmark	Europe	priv	619,000	10.69%	Bluetooth, Google/Apple	Y	Y	Y	Y	Y	Decentralized	Launched
HOIA	Estonia	Europe	priv	250,944	18.88%	Bluetooth, DP-3T, Google/Apple	Y			Y	Y	Decentralized	Launched
CareFiji	Fiji	Asia	gov	27,000	3.06%	Bluetooth	Y			Y	Y	Decentralized	Launched
Koronavilkku	Finland	Europe	gov, priv	2,500,000	45.31%	Bluetooth, Google/Apple	Y	Y	Y	Y	Y	Decentralized	Launched
TousAntiCovid	France	Europe	acad	2,400,000	3.58%	Bluetooth	Y	Y	Y	Y	Y	Centralized	Re-launched
Corona-Warn-App	Germany	Europe	priv	18,000,000	21.68%	Bluetooth, Google/Apple	Y	Y	Y	Y	Y	Decentralized	Launched
GH COVID-19 Tracker	Ghana	Africa	gov			Location	Y	N		N	N		Launched
Beat Covid Gibraltar	Gibraltar	Europe	priv	9000	26.69%	Bluetooth	Y	Y	Y	Y	Y	Decentralized	Launched
VirusRadar	Hungary	Europe	gov, priv	35,000	0.36%	Bluetooth	Y		Y	Y	Y	Centralized	Launched
Rakning C-19	Iceland	Europe	gov	140,000	38.45%	Location	Y	Y	Y	Y	Y	Both?	Launched
Aarogya Setu	India	Asia	gov	163,000,000	12.05%	Bluetooth, Location	N	Y	Y	N	N	Centralized	Launched
PeduliLindungi	Indonesia	Asia	gov	4,600,000	1.72%	Bluetooth, Location	Y	N	N	N	N	Centralized	Launched
AC19	Iran	Asia	acad	0	0.00%	NA	N	N	N	N	N		Launched
Covid Tracker	Ireland	Europe	gov	1,300,000	26.33%	Bluetooth, Google/Apple	Y	Y	Y	Y	Y	Decentralized	Launched
HaMagen 2.0	Israel	Asia	gov	2,000,000	22.51%	Location	N	Y	Y	Y	Y	Centralized	Launched
Immuni	Italy	Europe	priv	9,769,449	16.19%	Bluetooth, Google/Apple	Y	Y	Y	Y	Y	Decentralized	Launched
COCOA	Japan	Asia	gov	7,700,000	6.09%	Bluetooth, Google/Apple	Y	Y	Y	Y	Y	Decentralized	Launched
Shlonik	Kuwait	Asia	gov			Location	Y		N	N	N	Centralized	Launched
MyTrace	Malaysia	Asia	gov	100,000	0.32%	Bluetooth	Y	N	Y	N	N	Decentralized	Launched
CovidRadar	Mexico	America	gov	50,000	0.04%	Bluetooth	Y	N	N	N	N	Centralized	Launched
NZ COVID Tracer	New Zealand	Australia	gov	605,751	12.45%	Bluetooth, QR codes, Google/Apple	Y	Y	Y	N	Y	Centralized	Launched
StopKorona	North Macedonia	Europe	priv			Bluetooth	Y	Y	Y	Y	Y	Decentralized	Launched
StopCOVID NI	Northern Ireland	Europe	gov			Bluetooth, Google/Apple	Y					Decentralized	Launched
Smittestopp	Norway	Europe	gov	158,000	2.94%	Bluetooth, Google/Apple	Y	Y	Y	Y	Y	Decentralized	Launched
StaySafe	Philippines	Asia	priv	2,000,000	1.87%	Bluetooth	Y	N	N	N	N	Decentralized	Launched
ProteGO Safe	Poland	Europe	gov	725,000	1.91%	Bluetooth, Google/Apple	Y	Y	Y	Y	Y	Decentralized	Launched
Ehteraz	Qatar	Asia	gov	2,531,620	91.00%	Bluetooth, Location	N		N	N	N	Centralized	Launched
Tawakkalna	Saudi Arabia	Asia	gov	7,000,000	20.77%	Location	Y	N	Y	N	N	Centralized	Launched
Tabaud	Saudi Arabia	Asia	gov			Bluetooth, Google/Apple	Y	Y	Y	Y	N	Decentralized	Launched
TraceTogether	Singapore	Asia	gov	4,511,200	80.00%	Bluetooth, BlueTrace	N	N	Y	N	Y	Centralized	Launched
COVID Alert SA	South Africa	Africa	gov	600,000	1.0%	Bluetooth, Google/Apple	Y	Y	Y	Y		Decentralized	Launched
RadarCOVID	Spain	Europe	priv	7.033.536	17%	Bluetooth, DP-3T, Google/Apple	Y	Y		Y	Y	Decentralized	Launched
SwissCovid	Switzerland	Europe	acad	1,600,000	18.67%	Bluetooth, DP-3T, Google/Apple	Y	Y	Y	Y	Y	Decentralized	Launched
MorChana	Thailand	Asia	gov	3,690,000	53.15%	Bluetooth, Location	Y			N	N	Decentralized	Launched
E7mi	Tunisia	Asia	gov	23,140	0.20%	Bluetooth	Y	Y	Y	N	N	Centralized	Launched
Hayat Eve Sığar	Turkey	Asia	gov	14,186,000	17.30%	Bluetooth, Location	N	N	N	Y	N	Centralized	Launched
TraceCovid	UAE	America	gov			Bluetooth	N			Y	N	Decentralized	Launched
NHS COVID-19 App	UK	Europe	gov	19,000,000	28.51%	Bluetooth, Google/Apple	Y	Y	Y	Y	Y	Decentralized	Launched
BlueZone	Vietnam	Asia	gov	20,000,000	20.93%	Bluetooth	Y	Y	N	N	Y	Decentralized	Launched

**Table 2:** Configuration of $x_{i}\left(0\right)$ for counting. Nodes 1 to 6 mark with 1 the chosen option and the rest of the vector remains with zeros. An extra node $x_{0}$ is included, filled to zeros, and it is the only one that inits the auxiliary variable as one to count the number of participants in the process.

	$x_{i}^{1}$	$x_{i}^{2}$	$x_{i}^{3}$	| $y_{i}$
$x_{0}$	0	0	0	1
$x_{1}$	1	0	0	0
$x_{2}$	0	1	0	0
$x_{3}$	1	0	0	0
$x_{4}$	0	0	1	0
$x_{5}$	0	0	1	0
$x_{6}$	1	0	0	0
total	3	1	2	

**Table 3:** Sample of risk values by zone. *R* are the actual values, $R_{i}\left(0\right)$ the initial value for node *i*, and $R_{i}\left(t\right)$ the final values calculated by node *i*.

	1	2	3	4	5	6	7	8	9	10	11	12	13	14
$R_{i}\left(0\right)$	0	0	0	0	0	0	0	0	0	0	45	0	0	0
$R_{i}\left(t\right)$	10	8.1	12.3	8.6	13.2	9.5	49.2	48.3	40.1	12.5	22	9.6	11.4	8.8
*R*	9.9	10.1	9.9	9.9	12.2	11.3	50.3	55.6	53.1	15.4	21.8	9.9	10.9	10.1

## Data Availability

Publicly available datasets were analyzed in this study. This data can be found here: https://www.ine.es/en/experimental/movilidad/experimental_em_en.htm (accessed on 15 January 2021).
